# Prognostic Value of the TAPSE/sPAP Ratio in Patients with Type 2 Respiratory Failure: Insights into Right Ventricular–Pulmonary Arterial Coupling and Clinical Outcomes

**DOI:** 10.3390/diagnostics16111716

**Published:** 2026-06-03

**Authors:** Murat Karamanlıoğlu, Oral Menteş, Murat Yıldız, Ekrem Şahan, Maşide Arı, Vedat Kacar, Zeynep Büşra Biçer, Suzan Şahan

**Affiliations:** 1 Department of Cardiology, Ankara Atatürk Sanatorium Training and Research Hospital, University of Health Sciences, Ankara 06290, Türkiye; 2 Department of Intensive Care, Ankara Atatürk Sanatorium Training and Research Hospital, University of Health Sciences, Ankara 06280, Türkiye; omentes@live.com; 3 Department of Chest Diseases, Ankara Atatürk Sanatorium Training and Research Hospital, University of Health Sciences, Ankara 06290, Türkiye; drmuratyildiz85@gmail.com (M.Y.); masidetuten@icloud.com (M.A.); vedat0kacar@gmail.com (V.K.); zbb70707@gmail.com (Z.B.B.); 4 Department of Cardiology, Ankara Bilkent City Hospital, University of Health Sciences, Ankara 06800, Türkiye; ekremsahan@hotmail.com; 5 Department of Cardiology, Ankara Çubuk State Hospital, Ankara 06760, Türkiye; suzan_sahan@hotmail.com

**Keywords:** Type 2 respiratory failure, TAPSE/sPAP ratio, right ventricular function, pulmonary hypertension, mortality, echocardiography, RV–PA coupling, critical care

## Abstract

**Background/Objectives**: Type 2 respiratory failure (T2RF) is associated with significant morbidity and mortality, partly driven by cardiopulmonary interactions and right ventricular (RV) dysfunction. The tricuspid annular plane systolic excursion to systolic pulmonary artery pressure (TAPSE/sPAP) ratio has emerged as a non-invasive marker of RV–pulmonary arterial (RV–PA) coupling; however, its prognostic value in T2RF remains insufficiently explored. This study aimed to evaluate the association between TAPSE/sPAP and short-term clinical outcomes in hospitalized T2RF patients. **Methods**: In this retrospective cohort study, 182 adult patients hospitalized with T2RF between January 2024 and December 2025 were included. Patients were followed from hospital admission until discharge or death, and survival status was additionally evaluated up to 60 days after admission using hospital electronic medical records and follow-up databases for Kaplan–Meier survival analysis. Complete follow-up data were available for all included patients. Demographic, clinical, laboratory, and transthoracic echocardiographic data were analyzed. Patients were stratified into low and high TAPSE/sPAP groups. The primary outcome was in-hospital mortality; secondary outcomes included 60-day all-cause mortality, non-invasive ventilation (NIV) failure, intensive care unit (ICU) admission, and length of hospital stay. Statistical analyses included receiver operating characteristic (ROC) curves, multivariable logistic regression, calibration assessment, and decision curve analysis. **Results**: Patients with a low TAPSE/sPAP ratio had significantly higher in-hospital mortality (38.6% vs. 12.8%, *p* < 0.001), higher rates of NIV failure and ICU admission, and longer hospital stays. TAPSE/sPAP demonstrated the highest predictive performance for mortality (AUC: 0.82, 95% CI: 0.75–0.88), outperforming conventional echocardiographic parameters. In multivariable analysis, TAPSE/sPAP remained an independent predictor of mortality (OR: 1.48 per 0.1 decrease, *p* < 0.001). The model showed good calibration (Hosmer–Lemeshow *p* = 0.62), and decision curve analysis confirmed its clinical utility with a higher net benefit across a wide range of threshold probabilities. **Conclusions**: The TAPSE/sPAP ratio was independently associated with in-hospital mortality and adverse clinical outcomes in patients with T2RF, reflecting impaired RV–PA coupling. As a readily obtainable non-invasive echocardiographic parameter, it demonstrated promising prognostic value for risk stratification in this population. However, given the retrospective single-center design of the study, these findings should be considered hypothesis-generating and require confirmation in prospective multicenter studies before routine clinical implementation can be recommended.

## 1. Introduction

Type 2 respiratory failure (T2RF), also known as hypercapnic respiratory failure, is a complex clinical syndrome characterized by elevated arterial carbon dioxide tension (PaCO_2_ > 45 mmHg) resulting from alveolar hypoventilation [1,2]. It most commonly occurs in chronic respiratory disorders such as chronic obstructive pulmonary disease (COPD), obesity hypoventilation syndrome (OHS), bronchiectasis, and advanced neuromuscular diseases [3,4,5,6]. T2RF represents a critical condition associated with significant morbidity and mortality, particularly in hospitalized patients requiring advanced respiratory support, including non-invasive ventilation (NIV) or intensive care unit (ICU) admission [7,8].

In addition to chronic respiratory disorders, cardiovascular conditions may also contribute to the development or exacerbation of Type 2 respiratory failure. Congestive heart failure, pulmonary hypertension, right ventricular dysfunction, and acute cardiogenic pulmonary edema can impair gas exchange and increase the work of breathing, thereby promoting hypercapnia in susceptible individuals [1,2]. Furthermore, the close interaction between the respiratory and cardiovascular systems may amplify hemodynamic and ventilatory abnormalities, particularly in critically ill patients. These complex cardiopulmonary interactions highlight the importance of comprehensive cardiovascular assessment in patients with T2RF and support the investigation of prognostic markers reflecting right ventricular function and pulmonary vascular load.

The pathophysiological consequences of chronic hypoxemia and hypercapnia extend beyond pulmonary dysfunction and involve significant cardiovascular alterations. Persistent hypoxic pulmonary vasoconstriction leads to increased pulmonary vascular resistance, progressive elevation of systolic pulmonary artery pressure (sPAP), and ultimately right ventricular (RV) pressure overload [9,10,11,12,13,14]. Over time, these changes may result in right ventricular dysfunction, which is a well-established determinant of adverse outcomes in both acute and chronic respiratory diseases.

The assessment of RV function in patients with T2RF is clinically important but remains challenging due to the complex geometry and load dependency of the right ventricle. Conventional echocardiographic parameters such as tricuspid annular plane systolic excursion (TAPSE), RV fractional area change (FAC), and tissue Doppler-derived systolic velocity (S′) are widely used to evaluate RV systolic function [15,16,17,18,19]. However, these parameters alone may not fully reflect the interaction between the right ventricle and the pulmonary circulation, which plays a crucial role in disease progression and prognosis.

In this context, the ratio of TAPSE to sPAP has emerged as a novel, non-invasive echocardiographic index reflecting right ventricular–pulmonary arterial (RV–PA) coupling. This parameter integrates RV contractile function with afterload and provides a more comprehensive assessment of RV performance under varying hemodynamic conditions [20,21,22,23,24]. Impaired RV–PA coupling, indicated by a reduced TAPSE/sPAP ratio, has been associated with worse clinical outcomes, including increased mortality, in various cardiovascular and pulmonary conditions such as heart failure and pulmonary hypertension [25,26,27,28,29,30].

Recent guidelines from the European Society of Cardiology (ESC) and the European Respiratory Society (ERS) have recognized TAPSE/sPAP as an important prognostic marker and incorporated it into risk stratification models for pulmonary hypertension [31,32]. Despite this, the role of TAPSE/sPAP in patients with T2RF remains insufficiently explored. Particularly, there is a notable lack of data regarding its prognostic significance in heterogeneous populations such as COPD exacerbations and OHS-related respiratory failure.

Given that patients with T2RF frequently exhibit elevated pulmonary pressures and varying degrees of RV dysfunction, evaluating RV–PA coupling may provide valuable prognostic insights. Furthermore, early identification of high-risk patients may provide valuable prognostic information and support risk stratification in patients with T2RF.

Therefore, this study aims to investigate the prognostic value of the TAPSE/sPAP ratio and other echocardiographic right ventricular function parameters in adult patients hospitalized with Type 2 respiratory failure. Specifically, we evaluate their association with clinically relevant short-term outcomes, including in-hospital mortality, non-invasive ventilation failure, ICU requirement, and length of hospital stay.

## 2. Materials and Methods

### 2.1. Study Design

This study was designed as a single-center retrospective cohort study conducted at the Department of Chest Diseases Intensive Care Unit of Ankara Atatürk Sanatorium Training and Research Hospital, a tertiary referral center affiliated with the University of Health Sciences, Türkiye. The study protocol was reviewed and approved by the Institutional Scientific Research Ethics Committee (Approval No: 2024-BÇEK/520; Date: 8 April 2026). The study was conducted in accordance with the principles of the Declaration of Helsinki. The ethics committee approval specifically covered the retrospective review and analysis of anonymized medical records of patients hospitalized between 1 January 2024 and 31 December 2025, in accordance with institutional regulations and ethical guidelines for retrospective observational research. Written informed consent was obtained from all participants for participation in the study and publication of this paper.

Adult patients (≥18 years) hospitalized with a diagnosis of Type 2 respiratory failure (T2RF) between 1 January 2024 and 31 December 2025, were retrospectively screened for eligibility. T2RF was defined as hypercapnic respiratory failure characterized by an arterial partial pressure of carbon dioxide (PaCO_2_) > 45 mmHg on arterial blood gas analysis in conjunction with clinical findings consistent with alveolar hypoventilation. Eligible patients were identified through the hospital information management system, laboratory database, and digital imaging archive systems. Only patients with available transthoracic echocardiographic (TTE) examinations performed during routine clinical care were included in the study.

Patients were followed from hospital admission until hospital discharge, death, or completion of the 60-day follow-up period. Survival status at 60 days was obtained from hospital electronic medical records and institutional follow-up systems.

### 2.2. Inclusion and Exclusion Criteria

Patients were eligible for inclusion if they met all of the following criteria: age ≥ 18 years; hospitalization with a diagnosis of Type 2 respiratory failure; presence of hypercapnia defined as arterial PaCO_2_ > 45 mmHg on blood gas analysis; availability of transthoracic echocardiography performed within the first 24 h of hospital admission and before the occurrence of the primary outcome; and the ability to obtain measurable tricuspid annular plane systolic excursion (TAPSE) values along with a calculable systolic pulmonary artery pressure (sPAP) derived from the tricuspid regurgitation jet. Patients were excluded if they had hemodynamic instability requiring immediate endotracheal intubation at admission, known advanced pulmonary arterial hypertension, severe left-sided valvular heart disease, left ventricular systolic dysfunction defined as a left ventricular ejection fraction (LVEF) < 40%, congenital heart disease, inadequate echocardiographic imaging window, or missing essential clinical, laboratory, or echocardiographic data.

### 2.3. Data Collection Clinical Outcomes

All study data were retrospectively extracted from electronic medical records using a standardized case report form. Demographic and clinical variables included age, sex, and admission date, as well as underlying respiratory diseases such as chronic obstructive pulmonary disease (COPD), pneumonia, interstitial lung disease, bronchiectasis, and lung malignancy. In addition, comorbid conditions including diabetes mellitus, hypertension, chronic kidney disease, and heart failure were recorded.

Laboratory parameters obtained at admission or within the first 24 h of hospitalization were systematically collected. These included serum albumin, C-reactive protein (CRP), glucose levels, white blood cell (WBC) count, neutrophil and lymphocyte percentages, and platelet count. Renal function markers such as urea and creatinine, cardiac biomarkers including brain natriuretic peptide (BNP) and troponin, and coagulation-related parameters such as D-dimer were also documented. Electrolyte levels and liver function tests were recorded as part of routine biochemical evaluation.

Arterial blood gas analysis parameters, including pH, partial pressure of oxygen (PaO_2_), partial pressure of carbon dioxide (PaCO_2_), and bicarbonate (HCO_3_^−^), were obtained at admission or within the first 24 h and included in the analysis.

### 2.4. Echocardiographic Assessment

Transthoracic echocardiography (TTE) was performed using commercially available ultrasound systems (Vivid S60N, GE Healthcare, Horten, Norway) equipped with phased-array transducers. All examinations were performed by experienced board-certified cardiologists with at least 5 years of experience in echocardiographic imaging and interpretation, in accordance with current guideline recommendations [33,34]. All measurements were obtained as part of routine clinical evaluation and analyzed retrospectively. Right ventricular (RV) function was assessed using multiple echocardiographic parameters. Tricuspid annular plane systolic excursion (TAPSE) was measured in millimeters (mm) using M-mode echocardiography at the lateral tricuspid annulus. Systolic pulmonary artery pressure (sPAP) was expressed in mmHg and estimated from the peak tricuspid regurgitation velocity using the modified Bernoulli equation (4V^2^), with the addition of estimated right atrial pressure (RAP). RAP was assigned according to inferior vena cava diameter and inspiratory collapse, following current echocardiographic guideline recommendations.

The TAPSE/sPAP ratio was calculated as TAPSE (mm) divided by sPAP (mmHg), yielding units of mm/mmHg. In addition, right ventricular systolic velocity (S′) obtained by tissue Doppler imaging, RV fractional area change (FAC), and right ventricular chamber dimensions were recorded. Left ventricular systolic function was assessed by calculating the left ventricular ejection fraction (LVEF). The TAPSE/sPAP ratio was used as a non-invasive surrogate marker of right ventricular–pulmonary arterial (RV–PA) coupling, reflecting the relationship between RV contractile function and pulmonary vascular load. All echocardiographic measurements were obtained during routine clinical practice by experienced cardiologists and retrospectively reviewed by the study investigators.

To assess measurement reproducibility, a random sample of 30 echocardiographic examinations was re-evaluated. For intra-observer variability, the same cardiologist repeated the measurements after a 2-week interval while blinded to the initial results. For inter-observer variability, a second experienced cardiologist independently performed the measurements while blinded to the original measurements and clinical outcomes. Reproducibility was assessed using the intraclass correlation coefficient (ICC). The intra-observer ICCs were 0.94 for TAPSE and 0.92 for sPAP, whereas the inter-observer ICCs were 0.91 for TAPSE and 0.89 for sPAP, indicating excellent reproducibility.

### 2.5. Clinical Outcomes

The primary outcome of the study was in-hospital mortality. Secondary outcomes included 60-day all-cause mortality, non-invasive ventilation (NIV) failure, requirement for intensive care unit (ICU) admission, and length of hospital stay. Survival status at 60 days following hospital admission was obtained from hospital electronic medical records and follow-up databases. Follow-up data were available for all included patients, resulting in complete follow-up for survival analysis.

### 2.6. Statistical Analysis

All statistical analyses were performed using IBM SPSS Statistics version 27.0 (IBM Corp., Armonk, NY, USA) and R software (version 4.3.0; R Foundation for Statistical Computing, Vienna, Austria). The distribution of continuous variables was assessed using the Kolmogorov–Smirnov and Shapiro–Wilk tests, along with visual inspection of histograms and evaluation of skewness and kurtosis values. Normally distributed variables were expressed as mean ± standard deviation (SD), whereas non-normally distributed variables were presented as median with interquartile range (IQR). Categorical variables were summarized as frequencies and percentages [*n* (%)]. For comparative analyses, categorical variables were compared using the Chi-square test or Fisher’s exact test, as appropriate. Continuous variables were analyzed using Student’s *t*-test for normally distributed data or the Mann–Whitney U test for non-normally distributed variables. Patients were stratified into low and high TAPSE/sPAP groups according to the optimal cut-off value identified by receiver operating characteristic (ROC) curve analysis for predicting in-hospital mortality (≤0.34 vs. >0.34). Survival analysis was performed using the Kaplan–Meier method, and differences between groups were evaluated using the log-rank test. Receiver operating characteristic (ROC) curve analysis was conducted to assess the predictive performance of TAPSE/sPAP and other relevant variables for in-hospital mortality. The area under the curve (AUC) was calculated, and optimal cut-off values were determined using the Youden index. Sensitivity, specificity, positive predictive value (PPV), and negative predictive value (NPV) were also reported. Comparisons between ROC curves were performed using the DeLong test. To reduce the risk of model overfitting, only variables demonstrating clinical relevance and statistical significance in univariable analyses (*p* < 0.10) were considered for multivariable modeling. A backward stepwise elimination procedure was subsequently applied to derive the most parsimonious model. To identify independent predictors of in-hospital mortality, two separate multivariable logistic regression models were constructed: one including clinical, laboratory, and echocardiographic variables, and another including only echocardiographic parameters. A backward stepwise elimination method was applied to derive the most parsimonious models by sequentially removing non-significant variables. Model performance and explanatory power were evaluated using the Nagelkerke R^2^ coefficient. Given the biological relationship among several echocardiographic and laboratory variables, multicollinearity was carefully assessed using variance inflation factor (VIF) analysis. All variables included in the final models demonstrated acceptable VIF values (<2.5), indicating the absence of significant multicollinearity. To further minimize redundancy, composite indices such as the TAPSE/sPAP ratio were prioritized during model development. The calibration of the multivariable logistic regression models was assessed using the Hosmer–Lemeshow goodness-of-fit test and visually evaluated with calibration plots comparing predicted and observed probabilities of in-hospital mortality. Decision curve analysis (DCA) was performed to evaluate the clinical utility of the TAPSE/sPAP ratio and the multivariable models by quantifying the net benefit across a range of threshold probabilities for predicting in-hospital mortality. A two-tailed *p*-value of <0.05 was considered statistically significant, and all analyses were conducted with a 95% confidence interval (CI).

## 3. Results

**Figure 1:**
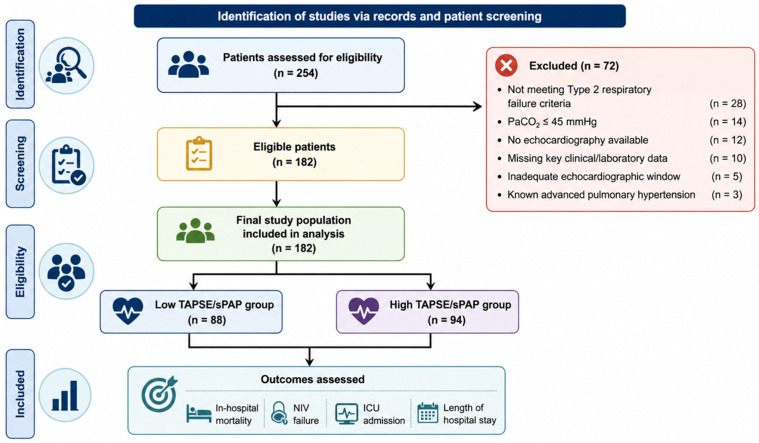
Flowchart of the study.

A total of 254 patients were initially assessed for eligibility. Among these, 72 patients were excluded due to not meeting the criteria for Type 2 respiratory failure (*n* = 28), PaCO_2_ ≤ 45 mmHg (*n* = 14), absence of echocardiographic data (*n* = 12), missing clinical or laboratory data (*n* = 10), inadequate echocardiographic imaging window (*n* = 5), and known advanced pulmonary hypertension (*n* = 3). The remaining 182 patients met the inclusion criteria and were included in the final analysis. The study population was stratified according to the TAPSE/sPAP ratio into two groups: low TAPSE/sPAP group (*n* = 88) and high TAPSE/sPAP group (*n* = 94). Clinical outcomes assessed in the study included in-hospital mortality, 60-day all-cause mortality, NIV failure, ICU admission, and length of hospital stay (Figure 1).

The study population consisted of 182 patients with a mean age of 68.4 ± 11.7 years, of whom 61.5% were male. Chronic obstructive pulmonary disease was the most common underlying respiratory condition (59.3%), followed by pneumonia (18.7%), bronchiectasis (8.2%), and interstitial lung disease (6.6%). The most frequent comorbidities were hypertension (52.7%) and diabetes mellitus (40.7%), while chronic kidney disease and heart failure were present in 21.4% and 23.1% of patients, respectively. Laboratory findings showed a mean albumin level of 3.2 ± 0.6 g/dL and a median CRP level of 38 mg/L (IQR: 18–74). The mean white blood cell count was 11.6 ± 4.2 ×10^9^/L with a neutrophil predominance (74.5 ± 9.8%) and reduced lymphocyte percentage (16.2 ± 6.7%). The median BNP level was 320 pg/mL (IQR: 145–780), and D-dimer levels were elevated with a median of 0.92 µg/mL (IQR: 0.48–1.76). Arterial blood gas analysis demonstrated acidemia with a mean pH of 7.31 ± 0.06, hypercapnia with a mean PaCO_2_ of 58.6 ± 11.4 mmHg, and reduced oxygenation with a mean PaO_2_ of 58.2 ± 12.5 mmHg (Table 1).

Echocardiographic evaluation of the study population demonstrated a mean TAPSE value of 17.8 ± 4.2 mm and a mean sPAP of 46.5 ± 12.8 mmHg. The median TAPSE/sPAP ratio was 0.38 (IQR: 0.28–0.51). Right ventricular systolic function parameters showed a mean RV S′ velocity of 10.2 ± 2.6 cm/s and a mean RV fractional area change of 37.6 ± 8.9%. Right ventricular dimensions were increased, with mean basal, mid, and longitudinal diameters of 41.8 ± 6.5 mm, 35.4 ± 5.9 mm, and 71.2 ± 8.3 mm, respectively. Moderate or greater tricuspid regurgitation was present in 37.4% of patients, and the estimated right atrial pressure was 9.6 ± 3.8 mmHg. Left ventricular systolic function was relatively preserved, with a mean LVEF of 55.8 ± 7.6%, while mild left ventricular systolic dysfunction (LVEF 40–49%) was observed in 13.2% of patients (Table 2).

Patients with a low TAPSE/sPAP ratio had significantly higher in-hospital mortality compared to those with a high TAPSE/sPAP ratio (38.6% vs. 12.8%, *p* < 0.001). The rates of non-invasive ventilation failure (46.6% vs. 19.1%, *p* < 0.001) and ICU admission (59.1% vs. 30.9%, *p* < 0.001) were significantly higher in the low TAPSE/sPAP group. The median length of hospital stay was also longer in this group (11 (7–17) days vs. 7 (5–11) days, *p* < 0.001). In terms of laboratory parameters, patients with a low TAPSE/sPAP ratio had higher CRP levels (52 vs. 29 mg/L, *p* = 0.002) and BNP levels (540 vs. 210 pg/mL, *p* < 0.001), as well as higher PaCO_2_ levels (61.2 ± 10.8 vs. 56.1 ± 11.5 mmHg, *p* = 0.010) and lower pH values (7.29 ± 0.05 vs. 7.33 ± 0.06, *p* < 0.001). Echocardiographic findings showed significantly lower TAPSE values (15.2 ± 3.8 vs. 20.3 ± 3.9 mm, *p* < 0.001) and higher sPAP levels (52.8 ± 13.6 vs. 40.3 ± 9.8 mmHg, *p* < 0.001) in the low TAPSE/sPAP group. RV fractional area change (33.1 ± 7.5% vs. 41.8 ± 7.2%, *p* < 0.001) and RV S′ velocity (8.9 ± 2.1 vs. 11.5 ± 2.3 cm/s, *p* < 0.001) were significantly reduced in this group (Table 3).

**Figure 2:**
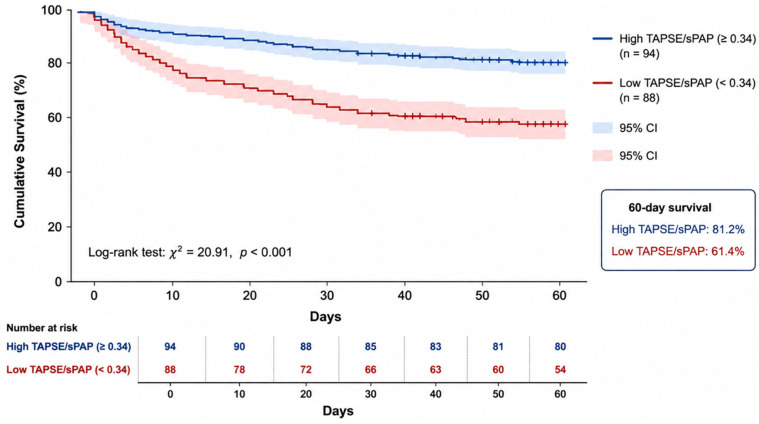
Kaplan–Meier survival curves according to TAPSE/sPAP ratio groups.

Kaplan–Meier survival analysis demonstrated that patients with a low TAPSE/sPAP ratio had significantly lower cumulative survival compared to those with a high TAPSE/sPAP ratio (log-rank test: χ^2^ = 20.91, *p* < 0.001). At 60 days, the survival rate was 61.4% in the low TAPSE/sPAP group and 81.2% in the high TAPSE/sPAP group (Figure 2).

**Figure 3:**
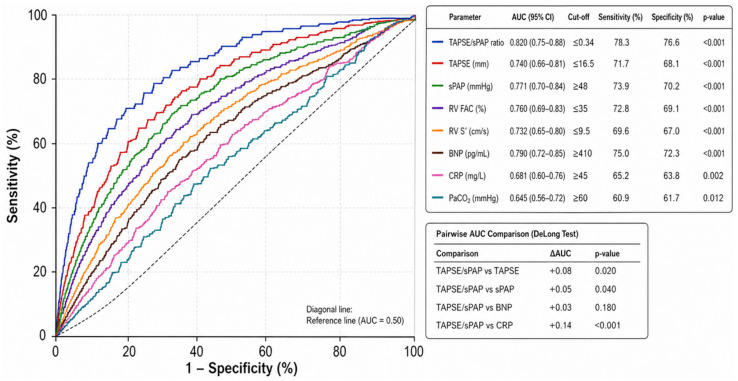
Receiver operating characteristic (ROC) curves for predicting in-hospital mortality.

ROC curve analysis demonstrated that the TAPSE/sPAP ratio had the highest predictive performance for in-hospital mortality, with an AUC of 0.820 (95% CI: 0.75–0.88, *p* < 0.001). The optimal cut-off value for TAPSE/sPAP was ≤0.34, yielding a sensitivity of 78.3% and specificity of 76.6%. Among other parameters, BNP (AUC: 0.790), sPAP (AUC: 0.771), and RV FAC (AUC: 0.760) also showed good predictive ability, while TAPSE (AUC: 0.740) and RV S′ (AUC: 0.732) demonstrated moderate performance. CRP (AUC: 0.681) and PaCO_2_ (AUC: 0.645) showed lower predictive accuracy. Pairwise comparisons using the DeLong test revealed that TAPSE/sPAP had significantly higher AUC values compared to TAPSE (ΔAUC = +0.08, *p* = 0.020) and sPAP (ΔAUC = +0.05, *p* = 0.040), while no significant difference was observed compared to BNP (*p* = 0.180). A significantly greater difference was observed when compared to CRP (ΔAUC = +0.14, *p* < 0.001) (Table 4, Figure 3).

No significant multicollinearity was detected among the variables included in the multivariable regression models, as all variables demonstrated acceptable VIF values (<2.5). In multivariable logistic regression analysis, the TAPSE/sPAP ratio emerged as an independent predictor of in-hospital mortality in both models. In Model 1, which included clinical, laboratory, and echocardiographic variables, a decrease in TAPSE/sPAP ratio was significantly associated with increased mortality risk (OR: 1.48, 95% CI: 1.22–1.81, *p* < 0.001). Other independent predictors included age (OR: 1.03 per year, *p* = 0.010), CRP (OR: 1.09 per 10 mg/L increase, *p* = 0.011), BNP (OR: 1.07 per 100 pg/mL increase, *p* < 0.001), and PaCO_2_ (OR: 1.12 per 5 mmHg increase, *p* = 0.022), while albumin showed a protective effect (OR: 0.64, *p* = 0.041). The model demonstrated good explanatory power (Nagelkerke R^2^ = 0.48) and calibration (Hosmer–Lemeshow *p* = 0.62). In Model 2, which included only echocardiographic parameters, the TAPSE/sPAP ratio remained a strong independent predictor (OR: 1.56, 95% CI: 1.29–1.89, *p* < 0.001), along with TAPSE (OR: 0.91, *p* = 0.011), sPAP (OR: 1.15 per 5 mmHg increase, *p* = 0.003), and RV FAC (OR: 0.95, *p* = 0.021). This model also showed acceptable performance (Nagelkerke R^2^ = 0.39) and calibration (Hosmer–Lemeshow *p* = 0.71) (Table 5).

**Figure 4:**
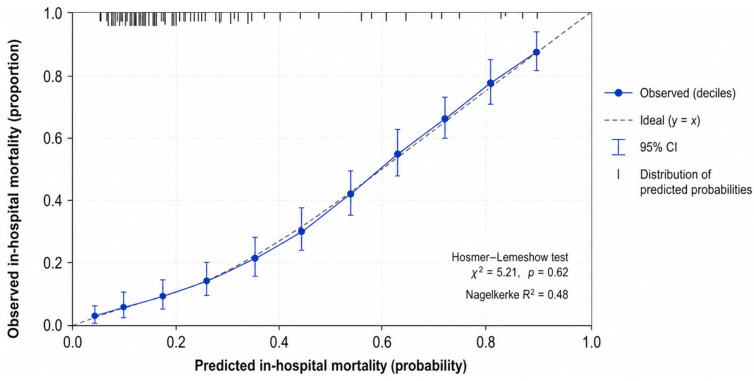
Calibration plot of the multivariable logistic regression model for predicting in-hospital mortality. Each point represents observed mortality (*y*-axis) versus predicted probability (*x*-axis) in each decile of risk. The dashed line represents perfect calibration. The model shows good agreement between predicted and observed outcomes across the range of predicted risks.

The multivariable logistic regression model demonstrated good calibration, as indicated by a non-significant Hosmer–Lemeshow test (*p* = 0.62). The calibration plot showed close agreement between predicted and observed in-hospital mortality across risk deciles, suggesting that the model was well-calibrated (Figure 4).

**Figure 5:**
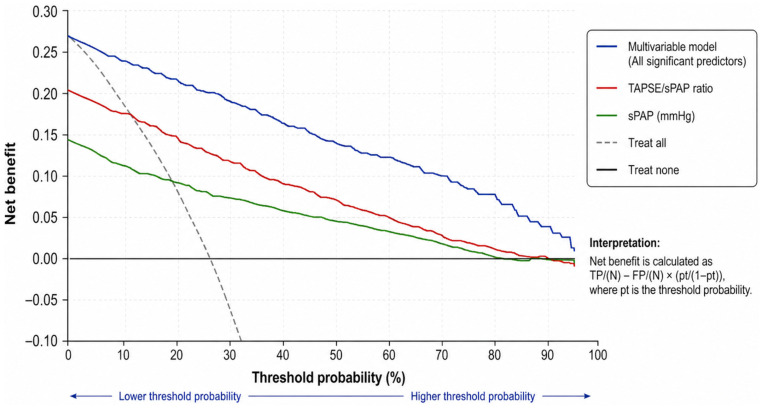
Decision curve analysis (DCA) for predicting in-hospital mortality. The decision curve shows the net benefit of using the model to predict in-hospital mortality across a range of threshold probabilities. The multivariable model (blue line) provides the highest net benefit across a wide range of threshold probabilities compared with the TAPSE/sPAP ratio alone, sPAP alone, and default strategies (treat all vs. treat none).

Decision curve analysis demonstrated that the TAPSE/sPAP ratio and the full multivariable model provided a higher net benefit across a wide range of clinically relevant threshold probabilities compared to both the treat-all and treat-none strategies (Figure 5).

A detailed comparison of disease severity characteristics, including respiratory comorbidities, pulmonary hypertension probability, baseline oxygenation and ventilation parameters, and treatment-related variables, is provided in Supplementary Table S1.

## 4. Discussion

In the current study, impaired RV–PA coupling, reflected by a lower TAPSE/sPAP ratio, was associated with adverse clinical outcomes in patients with Type 2 respiratory failure. Furthermore, TAPSE/sPAP demonstrated superior prognostic performance compared with several conventional echocardiographic parameters. These findings support the potential importance of RV–PA coupling assessment in this high-risk population.

The pathophysiological basis of our findings can be explained by the interplay between chronic hypoxemia, hypercapnia, and pulmonary vascular remodeling. In T2RF, sustained hypoxic pulmonary vasoconstriction increases pulmonary vascular resistance, leading to elevated sPAP and progressive RV afterload [9,10,11,12,13,14,15]. Over time, this results in RV dysfunction, which is a key determinant of adverse outcomes. Our findings support the concept that isolated RV functional indices may be insufficient, and that integrating RV contractility with afterload—as achieved by the TAPSE/sPAP ratio—provides a more comprehensive prognostic assessment.

Previous studies have consistently demonstrated the prognostic significance of RV–PA coupling in cardiovascular diseases. Guazzi et al. reported that impaired RV–PA coupling was strongly associated with adverse outcomes in patients with heart failure with preserved ejection fraction [20]. Kazimierczyk et al. showed that TAPSE/sPAP is a reliable predictor of mortality in pulmonary arterial hypertension patients [24]. In line with these findings, Fauvel et al. demonstrated that the TAPSE/sPAP ratio improves risk stratification in pulmonary hypertension and may outperform conventional parameters [30]. Our study extends these observations to a distinct and under-investigated population—patients with T2RF—highlighting that RV–PA uncoupling is also a critical determinant of prognosis in hypercapnic respiratory failure.

In terms of comparing individual echocardiographic parameters, we found that TAPSE/sPAP had a higher predictive accuracy than TAPSE or sPAP alone. This is consistent with the findings of Li et al., who emphasized that composite indices reflecting RV–PA interaction provide superior prognostic value compared to single parameters [23]. Schmeisser et al. demonstrated that TAPSE/sPAP correlates well with invasive pressure–volume loop-derived RV–PA coupling metrics, further supporting its physiological relevance [28]. Our ROC analysis confirmed this superiority, with TAPSE/sPAP achieving the highest AUC, and DeLong test comparisons showing statistically significant differences compared with several individual predictors.

Beyond discrimination, our study also evaluated calibration and clinical utility. The good agreement between predicted and observed outcomes in calibration analysis indicates that the model provides reliable risk estimation across different risk strata. Importantly, decision curve analysis demonstrated that both the TAPSE/sPAP ratio and the multivariable model offer a meaningful net clinical benefit over a wide range of threshold probabilities. These findings suggest that TAPSE/sPAP may have potential utility for risk stratification; however, prospective multicenter studies are required before its routine clinical application can be recommended.

Another notable finding of our study is the independent contribution of laboratory and clinical parameters such as CRP, BNP, PaCO_2_, and albumin levels. Elevated CRP reflects systemic inflammation, which is known to exacerbate pulmonary vascular dysfunction and RV impairment [9]. BNP is a well-established marker of ventricular strain and was significantly higher in patients with worse outcomes, consistent with previous studies [25]. Hypercapnia may further contribute to pulmonary vasoconstriction and acidosis, aggravating RV dysfunction. Conversely, hypoalbuminemia emerged as a protective factor, likely reflecting better nutritional and systemic status. These findings align with the growing evidence that both inflammatory and metabolic factors interact with cardiovascular dysfunction to influence outcomes in respiratory failure.

Our results are also consistent with studies focusing on RV function in critical illness. Al-Saadi et al. emphasized that RV dysfunction is a major but often under-recognized contributor to mortality in critically ill patients [35]. Hameed et al. highlighted the importance of comprehensive RV assessment, including coupling metrics, in predicting outcomes across various disease states [16]. In the context of T2RF, where pulmonary and cardiac interactions are particularly pronounced, our findings underscore the importance of integrating echocardiographic and clinical parameters for risk stratification.

Unlike most previously published studies that have evaluated the TAPSE/sPAP ratio primarily in cardiovascular populations such as heart failure and pulmonary hypertension, our study specifically focuses on patients with Type 2 respiratory failure—a clinically distinct group characterized by hypercapnia-driven pulmonary vascular and right ventricular alterations. While prior studies established the prognostic value of RV–PA coupling in cardiac settings, data in respiratory failure populations remain scarce. Moreover, our study not only confirms the prognostic relevance of TAPSE/sPAP in this setting but also demonstrates its incremental value over conventional echocardiographic and laboratory markers, supported by comprehensive analyses including ROC, calibration, and decision curve analysis. This integrative and clinically oriented approach provides novel evidence for the utility of TAPSE/sPAP in real-world respiratory critical care.

From a clinical perspective, the TAPSE/sPAP ratio offers several advantages. It is non-invasive, easily obtainable during routine echocardiography, and provides rapid bedside assessment. Given its strong association with mortality and clinical outcomes, it may serve as a valuable tool for early risk stratification. Patients with low TAPSE/sPAP ratios may represent a higher-risk subgroup; however, prospective studies are required before specific management implications can be recommended.

Some limitations of this study should be acknowledged. First, because only patients with available echocardiographic examinations were eligible for inclusion, selection bias cannot be completely excluded. Consequently, the study population may not fully represent all patients hospitalized with Type 2 respiratory failure. Second, echocardiographic measurements were obtained as part of routine clinical practice and may be subject to inter-observer variability. Third, only baseline measurements were analyzed, and dynamic changes in RV function over time were not evaluated. Fourth, although multivariable adjustment was performed, residual confounding cannot be entirely excluded. Although measures were taken to reduce model overfitting, including restricted variable selection and backward stepwise modeling, the relatively limited sample size may still have affected model stability. The study population included patients with heterogeneous etiologies of Type 2 respiratory failure, including COPD, pneumonia, bronchiectasis, and interstitial lung disease. Although additional analyses of disease severity characteristics were performed and are presented in Supplementary Table S1, residual confounding related to underlying disease severity and etiology cannot be entirely excluded. Additionally, the study was conducted at a single center, which may limit the generalizability of the findings. Finally, although advanced statistical methods such as ROC, calibration, and decision curve analysis were employed, external validation in independent cohorts is warranted. Therefore, the present findings should be considered hypothesis-generating rather than practice-changing. Although TAPSE/sPAP demonstrated promising prognostic performance, prospective multicenter studies with external validation are required before this parameter can be recommended for routine clinical decision-making or incorporated into management algorithms for patients with Type 2 respiratory failure.

## 5. Conclusions

In conclusion, the TAPSE/sPAP ratio was independently associated with adverse clinical outcomes and mortality in patients with Type 2 respiratory failure. As a non-invasive marker of right ventricular–pulmonary arterial coupling, it demonstrated promising prognostic value in this cohort. However, given the retrospective single-center design of the study, these findings should be considered hypothesis-generating. Further prospective multicenter studies with external validation are required to confirm the clinical utility of TAPSE/sPAP and determine its role in risk stratification strategies for patients with Type 2 respiratory failure.

## Figures and Tables

**Table 1:** Baseline demographic, clinical, and laboratory characteristics of the study population.

Variable	Overall (*n* = 182)
Age (years), mean ± SD	68.4 ± 11.7
Male sex, *n* (%)	112 (61.5%)
**Underlying Respiratory Diseases, *n* (%)**	
COPD	108 (59.3%)
Pneumonia	34 (18.7%)
Interstitial lung disease	12 (6.6%)
Bronchiectasis	15 (8.2%)
Lung malignancy	7 (3.8%)
Others	6 (3.3%)
**Comorbidities, *n* (%)**	
Hypertension	96 (52.7%)
Diabetes mellitus	74 (40.7%)
Chronic kidney disease	39 (21.4%)
Heart failure	42 (23.1%)
Others	28 (15.4%)
Albumin (g/dL), mean ± SD	3.2 ± 0.6
CRP (mg/L), median (IQR)	38 (18–74)
Glucose (mg/dL), mean ± SD	148 ± 52
WBC (×10^9^/L), mean ± SD	11.6 ± 4.2
Neutrophil (%)	74.5 ± 9.8
Lymphocyte (%)	16.2 ± 6.7
Platelet (×10^9^/L)	268 ± 96
BNP (pg/mL), median (IQR)	320 (145–780)
Troponin (ng/mL), median (IQR)	0.038 (0.012–0.091)
D-dimer (µg/mL), median (IQR)	0.92 (0.48–1.76)
Urea (mg/dL), mean ± SD	52 ± 28
Creatinine (mg/dL), mean ± SD	1.28 ± 0.6
Sodium (mmol/L)	136.8 ± 4.5
Potassium (mmol/L)	4.3 ± 0.6
AST (U/L), median (IQR)	28 (19–46)
ALT (U/L), median (IQR)	24 (16–39)
pH	7.31 ± 0.06
PaO_2_ (mmHg)	58.2 ± 12.5
PaCO_2_ (mmHg)	58.6 ± 11.4
HCO_3_^−^ (mmol/L)	29.8 ± 5.2

COPD: Chronic obstructive pulmonary disease; CRP: C-reactive protein; BNP: Brain natriuretic peptide; AST: Aspartate aminotransferase; ALT: Alanine aminotransferase; PaO_2_: Partial pressure of oxygen; PaCO_2_: Partial pressure of carbon dioxide; HCO_3_^−^: Bicarbonate.

**Table 2:** Echocardiographic parameters of the study population.

Parameter	Overall (*n* = 182)
**Right Ventricular Function**	
TAPSE (mm), mean ± SD	17.8 ± 4.2
sPAP (mmHg), mean ± SD	46.5 ± 12.8
TAPSE/sPAP ratio, median (IQR)	0.38 (0.28–0.51)
RV S′ velocity (cm/s), mean ± SD	10.2 ± 2.6
RV fractional area change (FAC, %), mean ± SD	37.6 ± 8.9
RV basal diameter (mm), mean ± SD	41.8 ± 6.5
RV mid diameter (mm), mean ± SD	35.4 ± 5.9
RV longitudinal diameter (mm), mean ± SD	71.2 ± 8.3
**Pulmonary Pressure & Valvular Findings**	
Tricuspid regurgitation (≥moderate), *n* (%)	68 (37.4%)
Estimated right atrial pressure (mmHg), mean ± SD	9.6 ± 3.8
**Left Ventricular Function**	
LVEF (%), mean ± SD	55.8 ± 7.6
Mild LV systolic dysfunction (LVEF 40–49%), *n* (%)	24 (13.2%)

TAPSE: Tricuspid annular plane systolic excursion; sPAP: Systolic pulmonary artery pressure; RV: Right ventricle; FAC: Fractional area change; LVEF: Left ventricular ejection fraction.

**Table 3:** Comparison of clinical outcomes according to TAPSE/sPAP ratio groups.

Variable	Low TAPSE/sPAP (*n* = 88)	High TAPSE/sPAP (*n* = 94)	*p*-Value
**Primary Outcome**			
In-hospital mortality, *n* (%)	34 (38.6%)	12 (12.8%)	<0.001
**Secondary Outcomes**			
NIV failure, *n* (%)	41 (46.6%)	18 (19.1%)	<0.001
ICU admission, *n* (%)	52 (59.1%)	29 (30.9%)	<0.001
Length of hospital stay (days), median (IQR)	11 (7–17)	7 (5–11)	<0.001
**Laboratory (selected)**			
CRP (mg/L), median (IQR)	52 (28–88)	29 (14–56)	0.002
BNP (pg/mL), median (IQR)	540 (260–1120)	210 (98–480)	<0.001
PaCO_2_ (mmHg), mean ± SD	61.2 ± 10.8	56.1 ± 11.5	0.010
pH	7.29 ± 0.05	7.33 ± 0.06	<0.001
**Echocardiography (selected)**			
TAPSE (mm)	15.2 ± 3.8	20.3 ± 3.9	<0.001
sPAP (mmHg)	52.8 ± 13.6	40.3 ± 9.8	<0.001
RV FAC (%)	33.1 ± 7.5	41.8 ± 7.2	<0.001
RV S′ (cm/s)	8.9 ± 2.1	11.5 ± 2.3	<0.001

NIV: Non-invasive ventilation; ICU: Intensive care unit; CRP: C-reactive protein; BNP: Brain natriuretic peptide; TAPSE: Tricuspid annular plane systolic excursion; sPAP: Systolic pulmonary artery pressure; RV: Right ventricle; FAC: Fractional area change.

**Table 4:** Receiver operating characteristic (ROC) curves for predicting in-hospital mortality.

Parameter	AUC (95% CI)	Cut-Off	Sensitivity (%)	Specificity (%)	*p*-Value
TAPSE/sPAP ratio	0.820 (0.75–0.88)	≤0.34	78.3	76.6	<0.001
TAPSE (mm)	0.740 (0.66–0.81)	≤16.5	71.7	68.1	<0.001
sPAP (mmHg)	0.771 (0.70–0.84)	≥48	73.9	70.2	<0.001
RV FAC (%)	0.760 (0.69–0.83)	≤35	72.8	69.1	<0.001
RV S′ (cm/s)	0.732 (0.65–0.80)	≤9.5	69.6	67.0	<0.001
BNP (pg/mL)	0.790 (0.72–0.85)	≥410	75.0	72.3	<0.001
CRP (mg/L)	0.681 (0.60–0.76)	≥45	65.2	63.8	0.002
PaCO_2_ (mmHg)	0.645 (0.56–0.72)	≥60	60.9	61.7	0.012
**Pairwise AUC Comparison (DeLong Test)**					
**Comparison**		**ΔAUC**	***p*-value**		
TAPSE/sPAP vs. TAPSE		+0.08	0.020		
TAPSE/sPAP vs. sPAP		+0.05	0.040		
TAPSE/sPAP vs. BNP		+0.03	0.180		
TAPSE/sPAP vs. CRP		+0.14	<0.001		

AUC: Area under the curve; CI: Confidence interval; TAPSE: Tricuspid annular plane systolic excursion; sPAP: Systolic pulmonary artery pressure; RV: Right ventricle; FAC: Fractional area change; BNP: Brain natriuretic peptide; CRP: C-reactive protein.

**Table 5:** Multivariable logistic regression analysis for predictors of in-hospital mortality.

**Model 1: Clinical + Laboratory + Echocardiographic Variables**			
**Variable**	**OR**	**95% CI**	***p*-value**
TAPSE/sPAP ratio (per 0.1 decrease)	1.48	1.22–1.81	<0.001
Age (per year)	1.03	1.01–1.06	0.010
CRP (per 10 mg/L increase)	1.09	1.02–1.16	0.011
BNP (per 100 pg/mL increase)	1.07	1.03–1.11	<0.001
PaCO_2_ (per 5 mmHg increase)	1.12	1.02–1.23	0.022
Albumin (per 1 g/dL increase)	0.64	0.42–0.97	0.041
**Model performance:** Nagelkerke R^2^ = 0.48; Hosmer–Lemeshow *p* = 0.62			
**Model 2: Echocardiographic Parameters Only**			
**Variable**	**OR**	**95% CI**	***p*-value**
TAPSE/sPAP ratio (per 0.1 decrease)	1.56	1.29–1.89	<0.001
TAPSE (mm)	0.91	0.84–0.98	0.011
sPAP (per 5 mmHg increase)	1.15	1.05–1.27	0.003
RV FAC (%)	0.95	0.91–0.99	0.021
**Model performance:** Nagelkerke R^2^ = 0.39; Hosmer–Lemeshow *p* = 0.71			

OR: Odds ratio; CI: Confidence interval; TAPSE: Tricuspid annular plane systolic excursion; sPAP: Systolic pulmonary artery pressure; RV: Right ventricle; FAC: Fractional area change; CRP: C-reactive protein; BNP: Brain natriuretic peptide.

## Data Availability

The datasets used and/or analyzed during the current study are available from the corresponding author upon reasonable request.

## Supplementary Materials

The following supporting information can be downloaded at: https://www.mdpi.com/article/10.3390/diagnostics16111716/s1. Table S1: Comparison of disease severity characteristics according to TAPSE/sPAP ratio groups.

## Abbreviations

T2RF	Type 2 respiratory failure
TAPSE	Tricuspid annular plane systolic excursion
sPAP	Systolic pulmonary artery pressure
RV	Right ventricle
RV–PA	Right ventricular–pulmonary arterial
FAC	Fractional area change
S′	Right ventricular systolic velocity (tissue Doppler)
LVEF	Left ventricular ejection fraction
NIV	Non-invasive ventilation
ICU	Intensive care unit
COPD	Chronic obstructive pulmonary disease
OHS	Obesity hypoventilation syndrome
CRP	C-reactive protein
BNP	Brain natriuretic peptide
WBC	White blood cell
PaO_2_	Partial pressure of oxygen
PaCO_2_	Partial pressure of carbon dioxide
HCO_3_^−^	Bicarbonate
ROC	Receiver operating characteristic
AUC	Area under the curve
CI	Confidence interval
DCA	Decision curve analysis
OR	Odds ratio
SD	Standard deviation
IQR	Interquartile range
VIF	Variance inflation factor
