# Supplementary material for: Prognostic Value of the TAPSE/sPAP Ratio in Patients with Type 2 Respiratory Failure: Insights into Right Ventricular–Pulmonary Arterial Coupling and Clinical Outcomes
Source: Diagnostics (Basel). 2026 Jun 3;16(11):1716. doi: 10.3390/diagnostics16111716 (PMC13256892; doi:10.3390/diagnostics16111716)
Supplement: Supplementary file 1 [file diagnostics-16-01716-s001.zip › diagnostics-4323337-supplementary.pdf]

Supplementary Table S1. Comparison of disease severity characteristics according to TAPSE/sPAP ratio groups.

**Variable**	**Low TAPSE/sPAP (n=88)**	**High TAPSE/sPAP (n=94)**	**p-value**
COPD, n (%)	58 (65.9)	50 (53.2)	0.082
COPD requiring long-term oxygen therapy, n (%)	34 (38.6)	22 (23.4)	0.028
Obesity hypoventilation syndrome (OHS), n (%)	18 (20.5)	11 (11.7)	0.104
Pneumonia, n (%)	20 (22.7)	14 (14.9)	0.177
Heart failure history, n (%)	28 (31.8)	14 (14.9)	0.007
Moderate/high probability of pulmonary hypertension, n (%)	49 (55.7)	23 (24.5)	<0.001
Baseline SpO_2 (%)	84.2 ± 6.8	88.5 ± 5.7	<0.001
PaO_2 (mmHg)	54.8 ± 11.6	61.3 ± 12.7	0.001
$PaCO_2$ (mmHg)	61.2 ± 10.8	56.1 ± 11.5	0.010
pH	7.29 ± 0.05	7.33 ± 0.06	<0.001
NIV use >48 h, n (%)	53 (60.2)	37 (39.4)	0.005
Initial ICU admission, n (%)	37 (42.0)	19 (20.2)	0.002
BNP (pg/mL), median (IQR)	540 (260–1120)	210 (98–480)	<0.001

COPD, chronic obstructive pulmonary disease; OHS, obesity hypoventilation syndrome; NIV, non-invasive ventilation; ICU, intensive care unit; BNP, brain natriuretic peptide.
